# Explaining the geographical origins of seasonal influenza A (H3N2)

**DOI:** 10.1098/rspb.2016.1312

**Published:** 2016-09-14

**Authors:** Frank Wen, Trevor Bedford, Sarah Cobey

**Affiliations:** 1 Department of Ecology and Evolution, University of Chicago, 1101 East 57th Street, Chicago, IL 60637, USA; 2 Vaccine and Infectious Disease Division, Fred Hutchinson Cancer Research Center, Seattle, WA, USA

**Keywords:** *R*_0_, viral migration, source–sink, molecular epidemiology

## Abstract

Most antigenically novel and evolutionarily successful strains of seasonal influenza A (H3N2) originate in East, South and Southeast Asia. To understand this pattern, we simulated the ecological and evolutionary dynamics of influenza in a host metapopulation representing the temperate north, tropics and temperate south. Although seasonality and air traffic are frequently used to explain global migratory patterns of influenza, we find that other factors may have a comparable or greater impact. Notably, a region's basic reproductive number (*R*_0_) strongly affects the antigenic evolution of its viral population and the probability that its strains will spread and fix globally: a 17–28% higher *R*_0_ in one region can explain the observed patterns. Seasonality, in contrast, increases the probability that a tropical (less seasonal) population will export evolutionarily successful strains but alone does not predict that these strains will be antigenically advanced. The relative sizes of different host populations, their birth and death rates, and the region in which H3N2 first appears affect influenza's phylogeography in different but relatively minor ways. These results suggest general principles that dictate the spatial dynamics of antigenically evolving pathogens and offer predictions for how changes in human ecology might affect influenza evolution.

## 1. Introduction

Antigenic variants of seasonal influenza continuously emerge and escape human immunity in a process known as antigenic drift. These drifted strains are less easily recognized by host immunity and therefore have a transmission advantage. More antigenically advanced strains are also more likely to spread globally and successfully perpetuate the evolutionary lineage of subsequent variants.

Asia has long been recognized as a major source of not only new influenza subtypes, but also new strains of seasonal influenza [1–4]. Influenza A/H3N2, A/H1N1 and two B lineages currently circulate in the human population, with the H3N2 subtype causing the most disease [5]. Phylogeographic analyses show that East, South and Southeast Asia contribute disproportionately to the evolution of seasonal H3N2, exporting most of the evolutionarily successful strains that eventually spread globally [6–10]. The trunk of H3N2's phylogeny traces the evolutionary path of the most successful lineage and was estimated to be located in Asia 87% of the time from 2000 to 2010 [10]. Additionally, strains of H3N2 isolated in East–Southeast Asia appear to be more antigenically advanced, with new antigenic variants emerging earlier in East–Southeast Asia than in the rest of the world [7,11]. These observations suggest that ecological differences between regions, such as climate and human demography, affect the local antigenic evolution of H3N2, which in turn shapes its global migratory patterns. Here, we ask what ecological factors might cause disproportionate contributions of particular host populations to the evolution of an influenza-like pathogen. This information may be immediately useful for viral forecasting. Over the long term, it could help predict changes in influenza's phylogeography and identify source populations to improve global vaccination strategies.

The conspicuous role of Asia in H3N2's evolution has been attributed to the seasonal nature of influenza in temperate regions [2,6–9,12]. Approximately 85% of Asia's population and 48% of the global population resides in a climatically tropical or subtropical region [13] where semiconnected host populations support asynchronous epidemics that enable regional persistence year-round [7,12,14]. Uninterrupted transmission might increase both the efficiency of selection and the probability of strain survival and global spread. By contrast, transmission bottlenecks from late spring through autumn in temperate populations necessarily limit local evolution and reduce opportunities for strain emigration [15,16]. Smaller contributions from other tropical and subtropical regions might arise from the weaker connectivity of their host populations [9,17,18].

Although seasonality clearly affects temporal patterns of viral migration [8], a robust explanation for differences in regions' long-term contributions to the evolution of H3N2 would consider the effects of seasonal variation in transmission of the light in other potentially influential differences among host populations, including as follows.

### (a) Host population size

East–South–Southeast Asia alone contains more than half of the global population [19]. Larger host populations should sustain larger viral populations, and in the absence of other effects, they should contribute a proportionally larger fraction of strains that happen to spread globally. Additionally, if rare mutations limit the generation of antigenic variants, larger populations could contribute a disproportionate number of antigenically novel strains with high fitness.

### (b) Host population turnover

Birth rates have historically been higher in East–South–Southeast Asia than in most temperate populations [19]. Demographic rates influence the replenishment of susceptibles and loss of immune individuals, thereby modulating selection for antigenic change. Faster replenishment of susceptibles increases prevalence, and thus viral abundance and diversity, but weakens the fitness advantage of antigenic variants. A more immune population imposes greater selection for antigenic change but supports a smaller, less diverse viral population. Thus, the rate of antigenic evolution may vary in a complex way with the rate of host population turnover [20].

### (c) Initial conditions

H3N2 first emerged in or near Hong Kong in 1968. The region in which a subtype emerges may effectively give the viral population a head start on evolution. The first epidemic will almost certainly occur in this region, and viruses here will be the first to experience selective pressure for antigenic change. If host migration rates are low and the founding viral population persists, this antigenic lead could be maintained or even grow in time.

### (d) Transmission rates

Differences in human behaviour can affect transmission rates. The transmission rate affects a strain's intrinsic reproductive number (*R*_0_), the expected number of secondary cases caused by a single infection in an otherwise susceptible population. Differences in regional *R*_0_ could affect evolution in at least two ways. Higher *R*_0_ increases the equilibrium prevalence, increasing the probability that rare beneficial mutations will appear. In addition, the rate of antigenic drift increases with *R*_0_ in models that include mutation as a diffusion-like process [10,21–23]. A higher intrinsic reproductive number in one population could thus accelerate the emergence of novel mutants in that area.

To understand the potential effects of these five factors on the evolution of H3N2 in space, we simulated an influenza-like pathogen in a simplified representation of the global human metapopulation. The simulated metapopulation consisted of three connected host populations, representing the temperate north, tropics and temperate south. Conceptually, the tropics in the model approximate Asia, where most of the population is tropical or subtropical [13] and epidemics are asynchronous, and exclude other less connected tropical and subtropical populations on other continents [9,17,18]. The two temperate populations approximate northern and southern populations where influenza is strongly seasonal. The model can also be generalized to represent three arbitrary populations by reducing seasonality.

**Figure 1.**
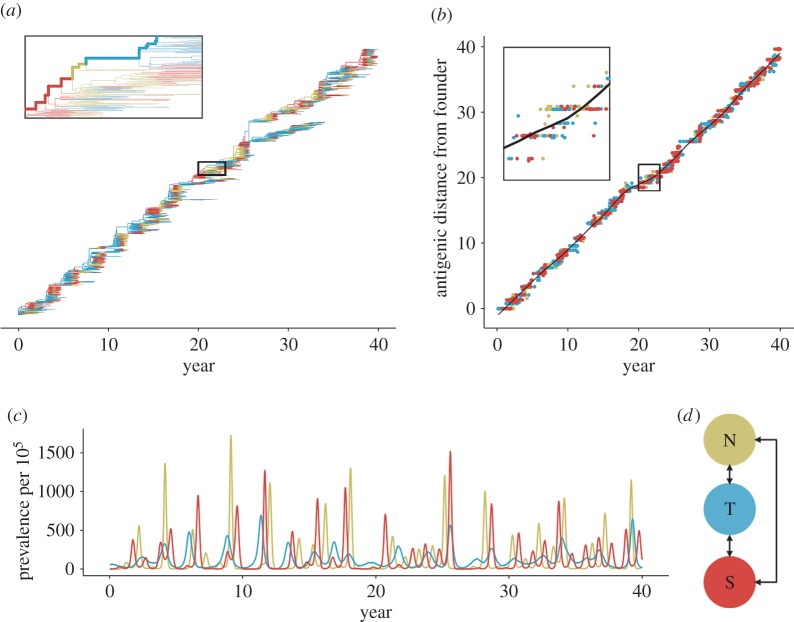
Representative output showing influenza-like behaviour from a sample simulation using the default parameters (table 2). Statistics reported here are based on 53 replicate simulations. (*a*) The phylogeny of the pathogen is reconstructed explicitly from the recorded ancestry of simulated strains. Branches are coloured by region indicated in panel (*d*). The trunk is determined by tracing the recorded ancestry of surviving strains at the end of the simulation. Side branches show lineages that go extinct. (*b*) Viruses evolve antigenically away from the founding strain in a canalized manner. On average, the antigenic distance from the founding strain follows the trajectory indicated by the black LOESS spline fitted to viruses from all three regions. At any given point in time, strains above this line have drifted farther from the founder compared with average, and are thus considered antigenically leading. Conversely, strains below this line are considered antigenically lagging. Antigenic lead is calculated as the distance to the spline in antigenic units. (*c*) Prevalence of infection over time for each region. (*d*) Depiction of the totally connected model population, composed of the temperate north, tropics and temperate south.

We analysed the effects of these factors on two key metrics of influenza's spatial evolutionary and antigenic dynamics. The first metric measures the proportion of the trunk of the phylogeny present in the tropics (figure 1*a*). The phylogenetic trunk represents the most evolutionarily successful lineage that goes on to seed all future outbreaks. The second metric measures the degree to which tropical strains are antigenically advanced (figure 1*b*). Phenotypically, antigenic dissimilarities can be quantified as distances in antigenic space using pairwise measures of cross-reactivity [11,24]. Our model uses an analogous measure of antigenic distances, allowing us to determine the relative antigenic advancement of strains from each region. We analysed these two metrics from simulations to test whether any of the five ecological factors could create spatial evolutionary patterns of a similar magnitude to the observed data.

## 2. Results

### (a) Influenza-like patterns

We simulated an individual-based model that included ecological and evolutionary dynamics in a metapopulation with three demes [25]. By default, in one deme, transmission rates are constant throughout the year, and in the two others, transmission rates vary sinusoidally with opposing phases. Viral phenotypes occur as points in two-dimensional Euclidean space, and mutation displaces phenotypes in this two-dimensional space according to a fixed kernel [25]. This space is analogous to an antigenic map constructed from pairwise measurements of cross-reactivity between influenza strains using a haemagglutination inhibition (HI) assay [11,24]. Susceptibility to infection is proportional to the distance in antigenic space between the challenging strain and the nearest strain in the host's infection history, giving distant or antigenically advanced strains greater transmissive advantage.

The model reproduces the characteristic ecological and evolutionary features of H3N2, except for the antigenic lead (table 1), under the default parameters (table 2). We restricted our analyses to simulations where the virus remained endemic and where the time to the most recent common ancestor (TMRCA) never exceeded 10 years during the 40 years of simulation. We chose this cut-off because in some simulations, the viral population developed unrealistically deep branches. In excluding extinctions and excessive diversity (branching), we assume that H3N2's historical evolutionary patterns represent the virus' likeliest evolutionary dynamics. Of 100 replicate simulations, the viral population went extinct in 18 cases and exceeded the TMRCA threshold 29 times, leaving 53 simulations for analysis. The model tracks the ancestry of individual strains, allowing us to explicitly reconstruct the phylogeny of the virus and the geographical location of lineages. The phylogeny has the characteristically well-defined trunk with short branches of the H3N2 haemagglutinin (figure 1). This shape arises due to repeated selective sweeps of antigenic variants, which reduces standing diversity; the average TMRCA across replicates was 3.72 years (s.d. = 0.26), comparable to empirical estimates of 3.89 years [10]. The antigenic distance from the founder increased linearly with time (figure 1), characteristic of H3N2's canalized antigenic evolution [24,25]. The mean antigenic drift across replicate simulations was 0.97 antigenic units per year (s.d. = 0.11), comparable to observed rates of 1.01 antigenic units per year [11]. The mean annual incidence was 9.1% (s.d. = 0.8%). Reported annual incidence across all subtypes of seasonal influenza ranges from 9% to 15% [26]. As we only modelled one lineage (e.g. the H3N2 subtype), the low estimate from the model is comparable to observed incidence.

**Table 1.** Properties of the default model.

statistic	model mean ± s.d.	observed (ref)
annual incidence	0.091 ± 0.0077	0.09–0.15 [26]
antigenic drift rate (antigenic units yr^–1^)	0.97 ± 0.11	1.01 [11]
TMRCA (years)	3.7 ± 0.26	3.89 [10]
fraction of trunk in the tropics	0.61 ± 0.13	0.87 [10]
tropics' antigenic lead (antigenic units)	0.0025 ± 0.036	0.25 [7,11]

**Table 2.** Default parameters.

parameter	value	reference
intrinsic reproductive number (*R*_0_)	1.8	[27,28]
duration of infection ν	5 days	[29]
population size *N*	45 million	(see the electronic supplementary material)
birth/death (turnover) rate *γ*	1/30 yr^–1^	[19]
mutation rate *μ*	10^−4^ d^−1^	(see the electronic supplementary material)
mean mutation step size *δ*_mean_	0.6 antigenic units	(see the electronic supplementary material)
s.d. mutation step size *δ*_s.d._	0.3 antigenic units	(see the electronic supplementary material)
infection risk conversion *c*	0.07	[25,30,31]
migration rate *m*	10^−3^ d^−1^	(see the electronic supplementary material)
seasonal amplitude *ε*	0.10	[32]

Although all three host populations were the same size, the tropical strains were on average more evolutionarily successful. The phylogenetic trunk traces the most evolutionarily successful lineage and was located in the tropics 77% (s.d. = 13%) of the time, comparable to the observed 87% of H3N2's trunk in East–South–Southeast Asia between 2000 and 2010 [10]. However, the default parametrization does not produce an antigenic lead in any population, despite the observed antigenic lead of Asian strains (table 1). Antigenic cartography shows that while H3N2 drifts on average at 1.01 antigenic units per year globally [11], Asian strains tend to be farther drifted at any given time, and the region is thus considered to lead antigenically [7,11].

### (b) Seasonality

**Figure 2.**
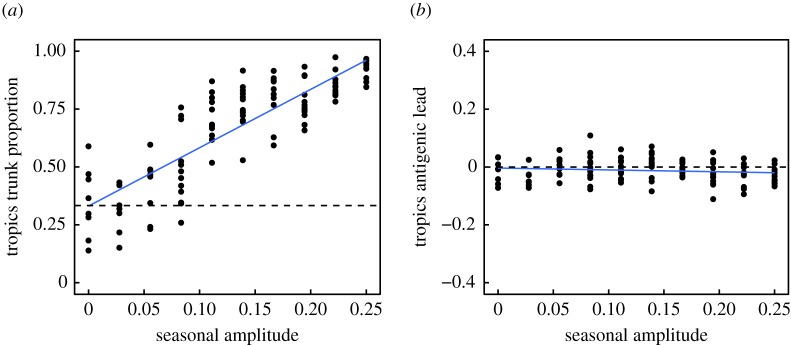
Seasonal amplitude *ε* in the temperate populations increases the tropics' contribution to the most evolutionarily successful lineage but alone does not affect regional differences in antigenic advancement. Transmission rates *β* in the temperate north and south oscillate sinusoidally in opposite phase, with amplitude *ε*. All other parameters remain at their default values (table 2). (*a*) Effects of seasonality on the fraction of the trunk in the tropics (Pearson's *r* = 0.85, *p* < 0.001; *R*^2^ = 0.72). Each point shows the fraction of time that the phylogenetic trunk was located in the tropics during the course of one simulation. The dashed line represents the null hypothesis where tropical strains comprise one-third of the phylogenetic trunk. (*b*) Effects on seasonality on the antigenic lead of the tropics (Pearson's *r* = −0.12, *p* = 0.20, *R*^2^ = 0.01). Each point shows the average antigenic lead of tropical strains over time from one simulation. The dashed line represents the null hypothesis where tropical strains are neither antigenically ahead or behind. Blue lines represent linear least-squares regression.

We first varied the strength of seasonal forcing, holding other parameters at their default values. Seasonality by itself in the two temperate populations could not cause the tropics to produce more antigenically advanced strains; however, seasonality did cause the tropics to contribute a greater fraction of evolutionarily successful strains (figure 2). By linear regression, we estimate that the trunk would spend 87% of its time in the tropics (the same fraction that is observed in Asia [10]) with a seasonal transmission amplitude (*ε*) of 0.19 (95% CI: 0.18, 0.20). Reduced seasonal forcing in the temperate populations equalized the fraction of the trunk in each population. In multivariate sensitivity analysis, the amplitude of seasonal transmission accounted for 33% of the variation in the tropical fraction of the trunk (electronic supplementary material, figure S2 and table S2). This result suggests that seasonal bottlenecks in temperate populations discourage seasonal strains from fixing globally, in agreement with other models [15]. However, seasonality alone could not explain any variation in the tropic's antigenic lead (electronic supplementary material, figure S2 and table S3). We therefore hypothesized that ecological factors besides seasonality must contribute to regional differences in relative antigenic fitness.

### (c) Transmission rate in the tropics

**Figure 3.**
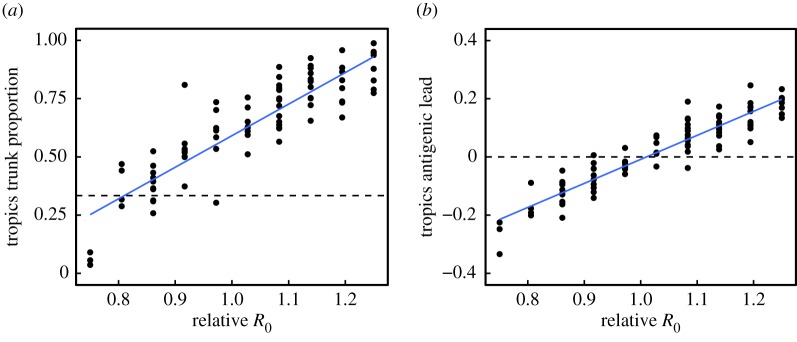
Increased *R*_0_ in the tropics increases the tropics' contribution to the most evolutionarily successful lineage and the antigenic advancement of tropical strains. Relative *R*_0_ is calculated as *R*_0_ in the tropics divided by *R*_0_ in the temperate regions. *R*_0_ in the tropics was varied while *R*_0_ in the temperate regions was kept at its default. Other parameters were also kept at their default values (table 2). (*a*) Effect of *R*_0_ in the tropics on the fraction of the trunk in the tropics (Pearson's *r* = 0.88, *p* < 0.001; *R*^2^ = 0.78). Each point shows the fraction of phylogenetic trunk located in the tropics during one simulation. The dashed line represents the null hypothesis where tropical strains comprise one-third of the phylogenetic trunk. (*b*) Effect of *R*_0_ in the tropics on the antigenic lead in the tropics (Pearson's *r* = 0.93, *p* < 0.001; *R*^2^ = 0.87). Each point shows the average antigenic lead of tropical strains over time from one simulation. The dashed line represents the null hypothesis where tropical strains are neither antigenically ahead or behind. Blue lines represent linear least-squares regression.

Increasing *R*_0_ in the tropics relative to the temperate populations caused the tropics to produce strains that led antigenically while also preserving the tropics' contribution to the trunk (figure 3). Linear regression implies that a 28% (95% CI: 25%, 30%) increase in *R*_0_ in the tropics causes the tropics to produce strains that are, on average, 0.25 antigenic units ahead of global mean, reproducing the observed antigenic lead in Asia [7,11]. We also estimate that a 17% increase in *R*_0_ (95% CI: 15%, 19%) causes the phylogenetic trunk to be located in the tropics 87% of the time, reproducing the observed fraction of the H3N2 trunk in Asia [10].

The effects of *R*_0_ on the antigenic lead were robust to changes in other ecological variables and over a range of baseline values of global *R*_0_. When we varied the other parameters (table 2), relative *R*_0_ in the tropics accounted for 77% of the variance in the antigenic lead, making it the best predictor of antigenic lead in the tropics (electronic supplementary material, figure S2 and table S3). The fraction of the trunk in the tropics also increased with the relative *R*_0_, although *R*_0_ explained less of the variation in trunk proportion (41%), due to the effect of seasonality (electronic supplementary material, figure S2 and table S2).

**Figure 4.**
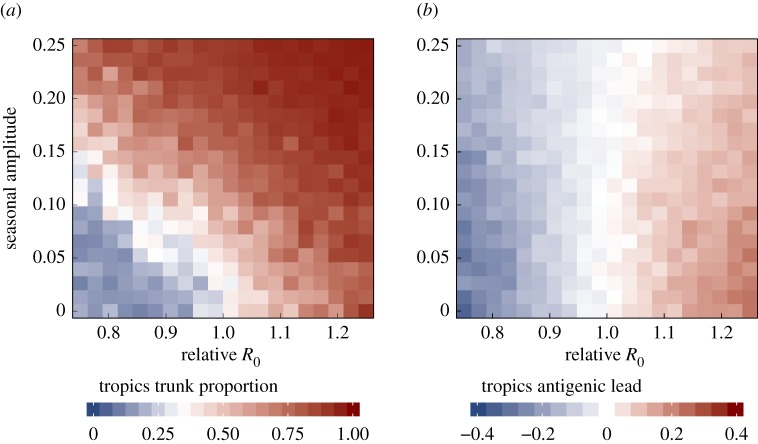
Seasonality in temperate populations has an equalizing effect on antigenic differences. Relative *R*_0_ is calculated as *R*_0_ in the tropics divided by *R*_0_ in the temperate regions. (*a*) Effects of seasonality and *R*_0_ on the fraction of the trunk in the tropics. Blue indicates that the phylogenetic trunk is located in the tropics less than one-third of the time, and red indicates that the trunk is in the tropics more than one-third of the time. (*b*) Effects of seasonality and *R*_0_ on antigenic lead in the tropics. Blue indicates that tropical strains are on average ahead antigenically relative to other global strains and red indicates that tropical strains are behind antigenically. Each square averages 1–17 replicate simulations.

Notably increased *R*_0_ in one deme was sufficient by itself to make strains more evolutionarily successful and antigenically advanced. When we removed seasonality altogether to model three climatically identical populations, the population with the highest *R*_0_ produced both the most antigenically leading and evolutionarily successful strains (figure 4). Thus, higher *R*_0_ alone in one region can cause it to attain an antigenic lead and fraction of the trunk as large as is observed in Asia.

To better understand why increasing regional *R*_0_ causes that region to produce more antigenically advanced strains, we examined the effect of *R*_0_ on antigenic evolution in a single deme. Simulations showed that increasing *R*_0_ increases the rate of antigenic drift (electronic supplementary material, figure S3). To investigate further, we derived an analytic expression for the invasion fitness of a novel mutant in a population at the endemic equilibrium (electronic supplementary material, equation (S1)). When the resident and mutant strains have the same intrinsic fitness (*R*_0_), the growth rate of an antigenically distinct, invading mutant increases linearly with *R*_0_ (electronic supplementary material, figure S4). This linearity holds as long as the conversion between antigenic distance and host susceptibility (equation (4.3)) is independent of *R*_0_. As *R*_0_ increases, not only do mutants invade faster, but also the invasion speed increases faster as a function of antigenic distance (electronic supplementary material, figure S4).

Although seasonality alone did not affect antigenic lead, the effects of *R*_0_ on antigenic lead could be influenced by seasonality (figure 4). Introducing seasonality in the temperate populations reduced differences in antigenic phenotype between regions. When tropical strains were antigenically ahead of temperate strains (due to higher tropical *R*_0_), introducing seasonality reduced the tropics' antigenic lead. When tropical strains were antigenically behind temperate strains (due to lower tropical *R*_0_), introducing seasonality reduced the antigenic lag. Two factors explain the equalizing effect of seasonality on antigenic phenotype. First, higher contact rates during transmission peaks in the two temperate populations increase the rate of strain immigration from the tropics. Second, seasonal troughs in prevalence allow tropical strains to invade more easily due to reduced competition with local strains.

### (d) Demographic rates, population size and initial conditions

Other ecological factors affected regional contributions to evolution but could not reproduce the observed patterns as well as differences in *R*_0_ (electronic supplementary material, figures S1 and S2). Notably, strains were slightly more antigenically advanced in older populations (electronic supplementary material, figure S1). When the rate of population turnover in the tropics was half that in the temperate regions, the tropics led by 0.04 antigenic units (s.d. = 0.03). Larger populations generally contributed more to the trunk, although there was much variation that population size alone did not explain (electronic supplementary material, figures S1, S2 and tables S2, S3). Initial conditions did not have a lasting effect (electronic supplementary material, figure S5).

### (e) Implications for other influenza subtypes

Both influenza A/H1N1 and influenza B evolve slowly compared with H3N2 and are suspected to have lower *R*_0_ [10,11]. Specifically, H1N1 drifts at a rate of 0.62 antigenic units per year, and the B/Victoria and Yamagata strains drift at 0.42 and 0.32 antigenic units per year, respectively [11]. H1N1 and B viruses are also less apt to have Asian origins than H3N2 [10]. When we simulate with lower baseline *R*_0_, we find that differences in *R*_0_ between regions have a weaker influence on spatial patterns of evolution (electronic supplementary material, figure S8). Based on the relationship between mean *R*_0_ and antigenic drift (electronic supplementary material, figure S3), we would expect seasonal H1N1, for example, to have an *R*_0_ of 1.6. For this *R*_0_, a 17% increase in *R*_0_ causes the tropics to occupy only 79% (versus 87% for H3N2-like *R*_0_ of 1.8) of the trunk, and a 28% increase in *R*_0_ causes the tropics to lead by 0.20 (versus 0.25 for H3N2) antigenic units.

## 3. Discussion

In our model, we find that the simplest explanation for why a host population produces more antigenically novel and evolutionarily successful strains than other populations is that its strains have a higher intrinsic fitness, or *R*_0_. The strong effect of regional *R*_0_ on spatial patterns of viral evolution is caused by the effect of *R*_0_ on antigenic drift. Higher regional *R*_0_ facilitates invasion of antigenically novel strains, resulting in faster antigenic drift. Seasonality reduces the rate at which temperate populations export strains that are evolutionarily successful, but seasonality alone cannot explain regional differences in the production of strains that are antigenically novel. Size and age can influence global patterns too, but to a lesser extent: larger populations export more strains that fix, and populations with slower replenishment of susceptibles increase the rate of antigenic evolution. These last two effects are sensitive to changes in seasonality and *R*_0_. These results highlight the relationship between human ecology and influenza's phylogeography. Regions with high transmission rates may be expected to contribute disproportionately to influenza's evolution and may also be ideal targets for vaccine campaigns. Accordingly, changes in human ecology can be expected to alter influenza's phylogeography. These generalizations assume that H3N2 will evolve mostly as it has, with high strain turnover and limited genetic variation at any time, but more complex dynamics may be possible.

To make general predictions, we used a simple model. Although our three-deme metapopulation prevents us from replicating influenza's phylogeographic dynamics precisely, the model nonetheless reveals how ecological differences in populations create spatial patterns in the evolution of an influenza-like pathogen. Simulations with more complex metapopulation models showed the same trends as the simple three-deme model (electronic supplementary material, figures S9 and S10), suggesting that our results are robust to changes in metapopulation population structure.

These results immediately raise the question of whether there is evidence of regional variation in *R*_0_. Low reporting rates and antigenic evolution make the *R*_0_ of influenza difficult to measure with traditional methods, but we can conjecture from several lines of evidence. Low absolute humidity favours transmission via aerosol in experimental settings [33] and influences the timing of the influenza season in the USA [34]. Based on absolute humidity and aerosol transmission alone, these results suggest that *R*_0_ of tropical and subtropical Asia would be lower than in temperate latitudes. However, in Vietnam the onset of influenza-like illness is associated with periods of high humidity [35]. This observation suggests that humidity is not the dominant driver of influenza transmission, at least in this region.

Contact rates also influence transmission [36]. Multiple studies have detected a significant effect of school closure on influenza spread [37–39], although this trend is not without exception [40]. Households also influence risk: after one household member is infected, the average risk of secondary infection in a household contact is 10% [41]. Differences in classroom and household sizes may thus influence local transmission, and both are higher in, for instance, China and India than in Europe and the USA [42,43]. Contact surveys report higher contact rates in Guangdong, China, than in European communities, whereas those in Vietnam are lower, although differences may arise from differences in survey design [44–46]. These surveys notably miss non-social, casual contacts (e.g. shared cafeterias and elevators) that might be important for influenza transmission.

Differences in local transmission rates may not scale: high rates of local transmission may be offset or attenuated by the structure of contact networks over larger areas. At the regional level, commuter and air passenger flows affect the spread of influenza epidemics, suggesting that adults are important to the long-range dispersal of the virus [12,18]. The frequency of long-distance contacts differs between communities [44]. Although sensitivity of *R*_0_ to network topology is well known theoretically [47,48], there is a need to integrate the features of local and regional empirical transmission networks to infer large-scale differences *R*_0_.

Empirical estimates of *R*_0_ are in theory attainable from seroprevalence. Under a simplistic, single-strain *SIR* model, which assumes random mixing and no maternal immunity, differences in *R*_0_ should appear in differences in seropositivity by age. For instance, if *R*_0_ = 1.8, approximately 5.1% of 2 year-olds would be seropositive, whereas 7.4% would be seropositive if *R*_0_ were 20% higher. *R*_0_ variation in this range could be detected by sampling as few as 1500 2-year-olds in each population. Detailed surveys of H3N2 seropositivity by age cohort exist for some European countries [49,50] but show much faster increases in seropositivity with age than expected under the *SIR* model: 100% of tested children are seropositive to H3N2 by age 7 in The Netherlands and by age 12 in Germany. This discrepancy between theory and data may be due to antigenic drift resulting in higher attack rates [10]. The spatial difference in seroprevalence may also reflect greater contact rates among school-aged children [45] and highlights the possibility that differences in exposure rates at young ages do not reflect mean differences in the populations. Such effects may be reduced by examining seroprevalence at older ages, but these estimates must balance a trade-off between minimizing age-related correlations in transmission rates and increasing sample sizes required to detect asymptotically small differences in seropositivity. Another potential approach to measuring *R*_0_ is to refine estimates of annual incidence in different populations. Estimates of *R*_0_ based on annual incidence would have to incorporate the histories of recent circulating strains, survey timing and titre dynamics and vaccination in each population.

A greatly reduced birth rate confers a slight antigenic lead, but actual differences in birth rates between regions appear too small to explain Asia's observed lead. Current birth rates across most of Europe, China and the USA are within 10% of each other [19]. Birth rates are almost twice as high in some Southeast Asian countries, including Cambodia, Laos and the Philippines. The highest birth rates are found in Africa and the Middle East, and are three to four times higher than birth rates in the USA and China. Our model suggests that these regions should contribute relatively less to influenza's antigenic evolution, assuming the differences in population structure are not associated with higher *R*_0_, and ignoring other differences. However, taking age-assortative mixing into account may negate this expectation, with younger populations having increased *R*_0_ [48,51] thus contributing more to antigenic evolution.

We expect these results to apply to other antigenically varying, fast-evolving pathogens, including other types of influenza. Enterovirus-71 circulates globally, and its VP1 capsid protein experiences continuous lineage replacement through time, similar to H3N2 haemagglutinin [52]. Norovirus also demonstrates rapid antigenic evolution by amino acid replacements in its capsid protein [53]. We might expect that areas with high transmission contribute disproportionately to the antigenic evolution and global spread of these pathogens. In addition, when we simulate with lower *R*_0_, we find that differences in *R*_0_ between regions influence spatial patterns of antigenic variation less (electronic supplementary material, figure S8). This may explain why influenza A H1N1 and influenza B, which are suspected to have lower *R*_0_ [10,11], are less apt to have Asian origins than H3N2 [10].

## 4. Material and methods

We implemented an individual-based *SIR* compartmental model of an influenza-like pathogen, originally described by Bedford *et al*. [25]. In this model, a global metapopulation is composed of three connected populations, representing tropics and temperate north and south. Individuals' compartments are updated using a *τ*-leaping algorithm. Within a region *i*, the force of infection is given by4.1
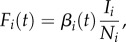
where *I* is the number of infected hosts. Between regions *i* and *j*, the force of infection is given by4.2
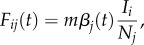
where region *i* is where the infection originates and region *j* is the destination. Here, *m* is a scaling factor for interregional transmission, and *β_j_* is the transmission rate of the destination region. Transmission rates in the seasonal north and south oscillate sinusoidally in opposite phase with amplitude *ε*. After recovery from infection, a host acquires complete immunity to viruses with that specific antigenic phenotype. Hosts that clear infection accumulate an infection history that defines their immunity. In a contact event, the distances between the infecting viral phenotype and each phenotype in the susceptible host's immune history are calculated. The probability of infection after contact is proportional to the distance *d* to the closest phenotype in the host's immune history. An individual's risk of infection by such a strain is4.3
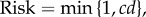
where the proportionality constant for converting antigenic distance to a risk of infection *c* = 0.07 [25]; in other words, one unit of antigenic distance corresponds to 7% reduction in immunity. The linear relationship *c* between antigenic distance and susceptibility derives from studies of vaccine efficacy [25,30,31].

Antigenic phenotypes are represented by points in a two-dimensional Euclidean antigenic space. One unit of antigenic distance in this space corresponds to a twofold dilution of antiserum in an HI assay [24]. The model is initialized at the endemic equilibrium with antigenically identical viruses. By default, all of the initial infections occur in the tropics. Mutational events occur at a rate *μ* mutations per day. When a virus mutates, it moves in a random radial direction with a gamma-distributed step size. This mutation rate, along with the mutation size parameters (*δ*subscript, *δ*_s.d._) determine the accessibility of more distant mutations in antigenic space. The radial direction of mutation is chosen from a uniform distribution.

## Supplementary Material

Electronic supplementary material

## Data accessibility

Additional methods are described in the electronic supplementary material. Code implementing the model is available at https://github.com/cobeylab/antigen-phylogeography.git. The complete code for reproducing these results is available at https://github.com/cobeylab/influenza_phylogeography_manuscript.git.
