# Supplementary material for: Explaining the geographical origins of seasonal influenza A (H3N2)
Source: Proc Biol Sci. 2016 Sep 14;283(1838):20161312. doi: 10.1098/rspb.2016.1312 (PMC5031657; doi:10.1098/rspb.2016.1312)
Supplement: Electronic supplementary material [file rspb20161312supp1.pdf]

Explaining the geographic origins of seasonal influenza A (H3N2): Electronic supplementary material

Frank Wen*[1], Trevor Bedford[2], and Sarah Cobey[1]

[1]Department of Ecology and Evolution, University of Chicago, 1101 E. 57th St., Chicago, IL 60637

[2]Vaccine and Infectious Disease Division, Fred Hutchinson Cancer Research Center

1 Extended methods

1.1 Selection of parameters

Parameter values were selected to be consistent with influenza's biology and to reproduce its major epidemiological and evolutionary patterns (table 1). The population size N was chosen to minimize extinctions while also making efficient use of computational resources. The population birth/death rate $\gamma = 1/30$ year^{-1} reflects the global crude birth rate estimates of 34 births per 1000 [1].

The proportionality constant m for calculating the between-region contact rate was calculated from the number of international air travel passengers reported by the International Civil Aviation Organization divided by the global population [2].

We chose a baseline $R_0 = 1.8$. Estimates of R_0 from the first pandemic wave H3N2 in 1968 range from 1.06-2.06 [3], and estimates of R_0 for seasonal influenza range from 1.16 to 2.5, averaging approximately 1.8 [4].

The five-day duration of infection, $1/\nu$, is based on estimates from viral shedding [5]. The transmission rate, β, is calculated using the definition of R_0:

$$R_0 = \frac{\beta}{\nu + \gamma}$$

We chose the seasonal amplitude to ensure consistent troughs during the off-season in temperate populations while remaining within reasonable estimates of seasonal transmission rates [6].

Mutational parameters were selected to maximize the number of simulations where evolution was influenza-like (figure S6). Mutations occur at a rate of 10^{-4} mutations per day. This phenotypic mutation rate corresponds to 10 antigenic sites mutating at 10^{-5} mutations per day [7, 8]. The distance of each mutation is sampled from a gamma distribution with parameters chosen to yield a mean step size of 0.6 and a standard deviation of 0.3 antigenic units. These values correspond to a reduction in immunity of 4.2% for an average mutation (SD = 2.1%). These mutation effect parameters give the gamma distribution an exponential-like shape, so that most mutations yield small differences in antigenic fitness, while occasionally mutations will yield greater differences. We chose μ, δ_{mean}, and δ_{sd} so that the simulations would exhibit influenza-like behaviour as consistently as possible (figure S6). Here, the criteria for influenza-like behavior included endemism, reduced genealogical diversity (TMRCA < 10 years) [9], and a biologically plausible mean rate of antigenic drift (1.01 antigenic units per year) [10] and incidence (9-15%) [11] (table 1, figure S6).

*frankwen@uchicago.edu

1.2 Calculation of antigenic lead and trunk proportion

We examined two metrics that describe influenza's evolutionary dynamics. For computational tractability, these metrics were calculated using a subset of strains sampled over course of the simulation. Strains were sampled proportionally to prevalence.

To calculate antigenic lead, we first calculated the antigenic distance of each sampled strain from the founding strain (figure 1*a*). We then fit a LOESS spline to these distances over time. The spline describes the expected antigenic drift of circulating lineages at any point in time. Strains above the spline have drifted farther than average and are considered antigenically leading. Strains below the spline have drifted less than average and are considered antigenically lagging. The antigenic lead in the tropics is calculated as the average antigenic distance to this spline for all sampled tropical strains.

To calculate the fraction of the trunk in each population, we first identified strains that comprise the trunk by tracing the lineage of strains that survive to the end of the simulation (figure 1*b*). Because multiple lineages may coexist at the end of the simulation, we excluded the last five years of strains from trunk calculations. The fraction of the trunk in the tropics is calculated as the fraction of the time the trunk was composed of tropical strains.

1.3 Univariate sensitivity analysis

In the univariate sensitivity analyses, we created regional differences in host ecology by varying each of the five ecological parameters individually (R_0, population turnover rate, seasonality, population size, and initial conditions) while keeping all other parameters at their default values (table 2). To test the effects of regional R_0, we changed R_0 only in the tropics. Similarly, we tested the effects of the rate of population turnover by varying it only in the tropics. To investigate seasonality, we varied the seasonal amplitude of the transmission rate in the temperate populations. (The transmission rate in the tropics was always constant over time.) To explore population size, we examined the ratio of tropical to temperate population sizes, keeping the global population constant. We initialized all simulations at the endemic equilibrium such that the total number of initial infecteds was constant. We then scaled the number of initial infecteds in the tropics while keeping the number in the two temperate demes the same. We ran twenty replicates for each unique combination of parameter values and discarded any simulations in which the virus went extinct or the TMRCA exceeded 10 years at any time in the 40-year simulation. The analyses for antigenic lead and trunk proportion were performed on the remaining simulations (figure S1).

1.4 Multivariate sensitivity analysis

To test the robustness of the effects of individual parameters on the antigenic lead and the phylogenetic trunk, we simulated 500 points from a Latin hypercube with dimensions representing relative R_0, seasonality, relative population size, relative population turnover rate, and the fraction of initial infecteds in the tropics (figure S2). The ranges for each parameter (table S1) were chosen to remain within reasonable estimates. We simulated twenty replicates for each of the 500 unique parameter combination and discarded simulations in which the virus went extinct or the TMRCA exceeded 10 years at any time in the 40-year simulation. We performed an ANOVA on the remaining 4119 influenza-like simulations to determine each parameter's contribution to the variance in antigenic lead and the fraction of the trunk in the tropics (table S2, S3).

2 Supporting Information

2.1 Invasion analysis

We assume that the host population supports a resident strain at the endemic equilibrium. We develop an expression for the fitness of an invading mutant strain to explain how the selection coefficient of the mutant changes with R_0.

Here, S, I, and R represent the fraction of susceptible, infected, and recovered individuals. The birth rate γ

and the death rate are equal, so the population size is constant. All individuals are born into the susceptible class. Transmission occurs at rate β, and recovery occurs at rate ν.

$$\frac{dS}{dt} = \gamma(1-S) - \beta SI$$
$$\frac{dI}{dt} = \beta SI - (\nu+\gamma)I$$
$$\frac{dR}{dt} = \nu I - \gamma R$$

We solve for the endemic equilibrium values of S_{eq}, I_{eq}, R_{eq}.

$$\frac{dI}{dt} = 0 = \beta S_{\text{eq}} I_{\text{eq}} - (\nu+\gamma) I_{\text{eq}}$$
$$S_{\text{eq}} = \frac{\nu+\gamma}{\beta} \equiv \frac{1}{R_0}$$

R_0, the basic reproductive number, is defined as the number of secondary infections from a single infected individual in a totally susceptible population. Continuing to solve for I_{eq} and R_{eq}, we have

$$\frac{dS}{dt} = 0 = \gamma(1-S_{\text{eq}}) - \beta S_{\text{eq}} I_{\text{eq}}$$
$$I_{\text{eq}} = \frac{\gamma}{\beta}(R_0 - 1)$$
$$\frac{dR}{dt} = 0 = \nu I_{\text{eq}} - \gamma R_{\text{eq}}$$
$$R_{\text{eq}} = \frac{\nu}{\beta}(R_0 - 1)$$

To find the selection coefficient, we develop an expression for the effective reproductive number R_e for both the resident and mutant strains. R_e is the expected number of secondary infections from a single infected individual in a given population. We will use the relationship

$$R_e = SR_0$$

The mutant strain is d antigenic units from the resident strain. The conversion factor between antigenic units and infection risk is notated by c. Thus, the susceptibility to the mutant is given by $\min\{cd, 1\}$, and immunity to the mutant is $\max\{1-cd, 0\}$. For ease of notation, we assume $cd \leq 1$, and use $k = 1 - cd$. The fraction of the population immune to the invading strain is denoted by R'. Note that the population is at the endemic equilibrium of the resident strain, and not the mutant.

$$R' = (1-cd)R_{\text{eq}}$$
$$= \frac{\nu}{\beta}(R_0 - 1)k$$

We start by allowing coinfection. The fraction of susceptibles to the mutant strain is given by

$$S' = 1 - R' - \frac{1}{N}$$
$$= 1 - \frac{\nu k}{\beta}(R_0 - 1) - \frac{1}{N}$$

For large N, we have

$$S' = 1 - \frac{\nu k}{\beta}(R_0 - 1)$$

As defined by our initial set of ODEs, the growth rates of the mutant and resident strains are

$$\begin{aligned}\frac{dI'}{dt} &= I'[\beta S' - (\nu + \gamma)] \\ \frac{dI}{dt} &= I_{\text{eq}}[\beta S_{\text{eq}} - (\nu + \gamma)]\end{aligned}$$

To get the selection coefficient, we take the difference between the growth rates:

$$\begin{aligned}s &= [\beta S' - (\nu + \gamma)] - [\beta S_{\text{eq}} - (\nu + \gamma)] \\ &= \beta - \gamma k(R_0 - 1) - \frac{\beta}{R_0}\end{aligned}$$

Recall that $\beta = (\nu + \gamma)R_0$

$$s = (\nu + \gamma)R_0 - \nu k(R_0 - 1) - (\nu + \gamma)$$

Simplifying,

$$s = (\nu cd + \gamma)(R_0 - 1) \tag{S1}$$

Now disallowing coinfection, we have

$$\begin{aligned}S' &= 1 - R' - I_{\text{eq}} - I' \\ &= 1 - \frac{\nu}{\beta}(R_0 - 1)k - \frac{\gamma}{\beta}(R_0 - 1) - \frac{1}{N}\end{aligned}$$

For large N,

$$S' = 1 - (R_0 - 1)[\frac{\nu k + \gamma}{\beta}]$$

Using the same arithmetic as in the case with coinfection, it follows that

$$s = \beta - (\nu k + \gamma)(R_0 - 1) - \frac{\beta}{R_0}$$

Simplifying,

$$s = (\nu cd)(R_0 - 1) \tag{S2}$$

In summary, the selection coefficient of an invading mutant strain increases linearly with the R_0, which is shared by both strains. The slope of this relationship is proportional to the distance d between the two strains in antigenic space (figure S4). Naturally, relationship between the selection coefficient on the distance d between strains depends on the functional relationship between antigenic distance and immunity. However, the linear dependence of the selection coefficient on R_0 holds as long as the functional relationship between antigenic distance and immunity is independent of R_0.

2.2 Detecting differences in R_0

In the SIR model, the force of infection is

$$F = \beta I$$

where β is the transmission rate and I is the fraction of infecteds. At the endemic equilibrium, the cumulative fraction of seropositive individuals at a given age a is

$$\begin{aligned} f(a) &= 1 - \exp(-\beta I_{\text{eq}} a) \\ &= 1 - \exp(-R_0(\nu + \gamma I_{\text{eq}} a) \end{aligned}$$

where ν is the recovery rate and γ is the birth/death rate. I_{eq}, the fraction of infecteds at the endemic equilibrium, is given by

$$I_{\text{eq}} = \frac{\gamma}{\beta}(R_0 - 1)$$

Figure S7 shows the fraction of seropositive individuals by age for the baseline $R_0 = 1.8$ and a 20% higher $R_0 = 2.16$. The difference in the percentage of seropositive two-year-olds between the two groups is approximately 2.3%. The sample size in each group required to detect a difference $f_2(a) - f_1(a)$ with α confidence and $1 - \beta$ power is

$$N = \frac{f_1(1 - f_1) + f_2(1 - f_2)}{(f_1 - f_2)^2}(\Phi_{\alpha/2} + \Phi_\beta)^2$$

For legibility, $f_i(a)$ is written as f_i. To detect a 20% difference in R_0 between two populations with 0.05 significance and 0.80 power, we would require a sample of at least 1503 individuals in both groups.

References

[1] World Population Prospects: The 2012 Revision. New York; 2013.

[2] Annual report of the ICAO council: 2014; 2014. Available from: http://www.icao.int/annual-report-2014/Pages/default.aspx.

[3] Jackson C, Vynnycky E, Mangtani P. Estimates of the transmissibility of the 1968 (Hong Kong) influenza pandemic: evidence of increased transmissibility between successive waves. American Journal of Epidemiology. 2009 Dec;171(4):465–478. Available from: http://dx.doi.org/10.1093/aje/kwp394.

[4] Biggerstaff M, Cauchemez S, Reed C, Gambhir M, Finelli L. Estimates of the reproduction number for seasonal, pandemic, and zoonotic influenza: a systematic review of the literature. BMC Infect Dis. 2014;14(1):480. Available from: http://dx.doi.org/10.1186/1471-2334-14-480.

[5] Carrat F, Vergu E, Ferguson NM, Lemaitre M, Cauchemez S, Leach S, et al. Time lines of infection and disease in human influenza: a review of volunteer challenge studies. American Journal of Epidemiology. 2008 Mar;167(7):775–785. Available from: http://dx.doi.org/10.1093/aje/kwm375.

[6] Truscott J, Fraser C, Cauchemez S, Meeyai A, Hinsley W, Donnelly CA, et al. Essential epidemiological mechanisms underpinning the transmission dynamics of seasonal influenza. Journal of The Royal Society Interface. 2011 Jun;9(67):304–312. Available from: `http://dx.doi.org/10.1098/rsif.2011.0309`.

[7] Rambaut A, Pybus OG, Nelson MI, Viboud C, Taubenberger JK, Holmes EC. The genomic and epidemiological dynamics of human influenza A virus. Nature. 2008 Apr;453(7195):615–619. Available from: `http://dx.doi.org/10.1038/nature06945`.

[8] Bedford T, Rambaut A, Pascual M. Canalization of the evolutionary trajectory of the human influenza virus. BMC Biology. 2012;10(1):38. Available from: `http://dx.doi.org/10.1186/1741-7007-10-38`.

[9] Bedford T, Riley S, Barr IG, Broor S, Chadha M, Cox NJ, et al. Global circulation patterns of seasonal influenza viruses vary with antigenic drift. Nature. 2015 Jun;523(7559):217–220. Available from: `http://dx.doi.org/10.1038/nature14460`.

[10] Bedford T, Suchard MA, Lemey P, Dudas G, Gregory V, Hay AJ, et al. Integrating influenza antigenic dynamics with molecular evolution. eLife. 2014 Feb;3. Available from: `http://dx.doi.org/10.7554/eLife.01914`.

[11] World Health Organization. Influenza Fact Sheet; 2014.

3 Supplemental tables and figures

Table S1: Parameter ranges used in Latin hypercube sampling

Parameter	Range
Relative R_0	0.8−1.2
Seasonal amplitude (ϵ) in temperate populations	0.0−0.15
Relative population size (N)	0.5−2.0
Relative turnover rate (γ)	0.5−2.0
Fraction of initial infecteds (I_0) in tropics	0.0−1.0

Table S2: ANOVA of the fraction of trunk in tropics from multivariate sensitivity analysis

Parameter	Df	Sum Sq	Frac of var	Mean Sq	F value	Pr(>F)
Relative N	1	5.04	0.017	5.04	316.48	<0.0001
Fraction I_0 in tropics	1	0.62	0.002	0.62	38.94	<0.0001
Relative R_0	1	114.49	0.406	114.49	7193.84	<0.0001
Relative turnover	1	2.37	0.008	2.37	148.97	<0.0001
Seasonal amplitude	1	94.04	0.334	94.04	5908.50	<0.0001
Residuals	4109	65.40	0.232	0.02		
Total	4114	281.96	1.000			

Parameter	Df	Sum Sq	Frac of var	Mean Sq	F value	Pr(>F)
Relative N	1	1.94	0.033	1.94	754.71	<0.0001
Fraction I_0 in tropics	1	0.02	<0.001	0.02	8.75	0.0031
Relative R_0	1	44.55	0.766	44.55	17344.41	<0.0001
Relative turnover	1	1.04	0.018	1.04	406.81	<0.0001
Seasonal amplitude	1	0.02	<0.001	0.02	9.53	0.0020
Residuals	4109	10.56	0.182	0.00		
Total	4114	58.140	1.000			

Table S3: ANOVA of the tropics' antigenic lead from multivariate sensitivity analysis

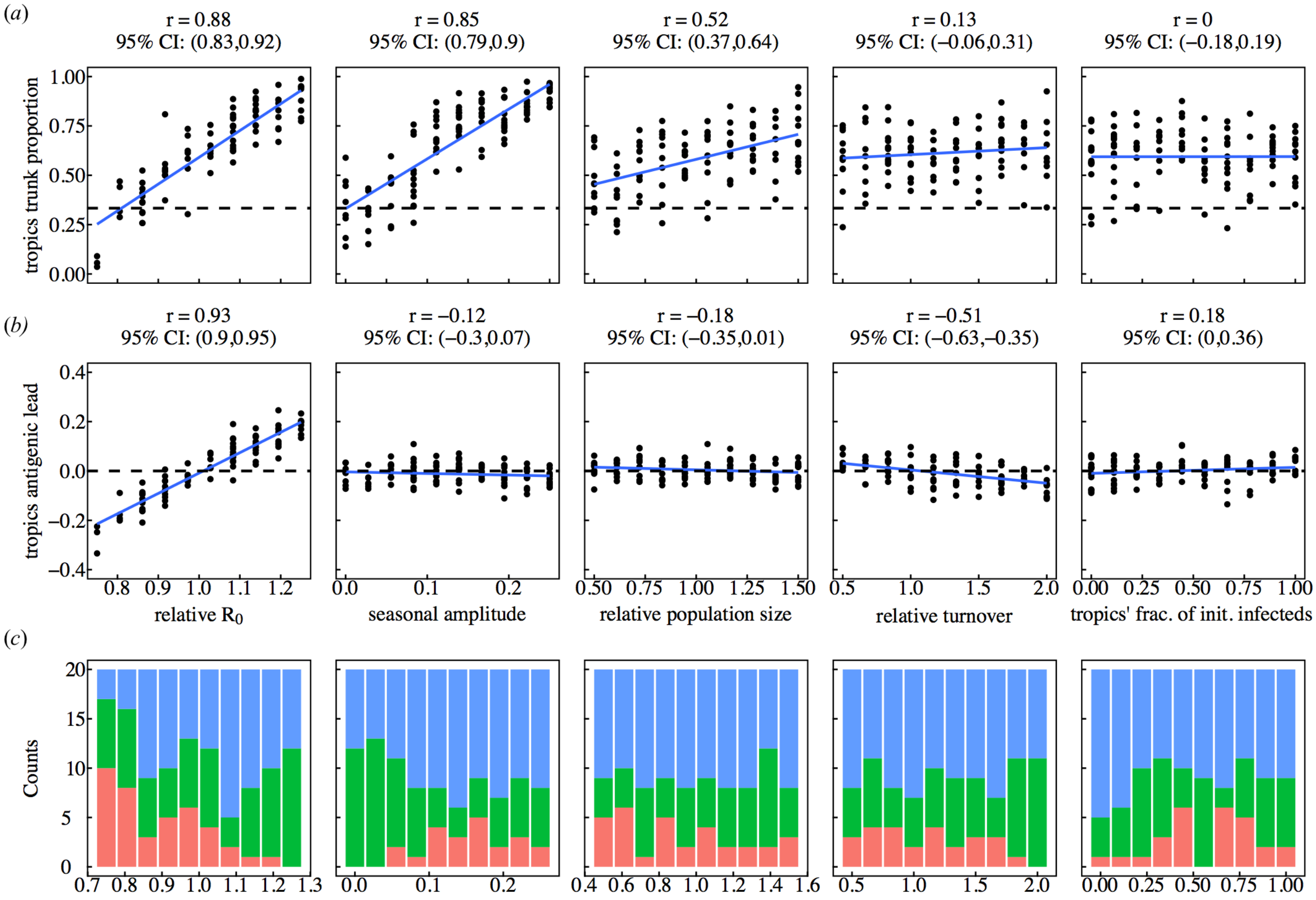

Figure S1: Univariate sensitivity analysis showing effects of individual parameters on (*a*) the antigenic lead and (*b*) the fraction of the phylogenetic trunk in the tropics. In each column of plots, only the parameter indicated on the x-axis is varying; all others are held constant at the default value. Each point represents the mean value over a single simulation. Blue lines indicate linear least squares regression. The dashed lines represent the null hypotheses where (*a*) the trunk is distributed equally among the three regions or (*b*) tropical strains are neither antigenically ahead or behind. (*c*) Number of simulations that went extinct (red), exceeded the TMRCA limit (green), or were suitable for analysis (blue).

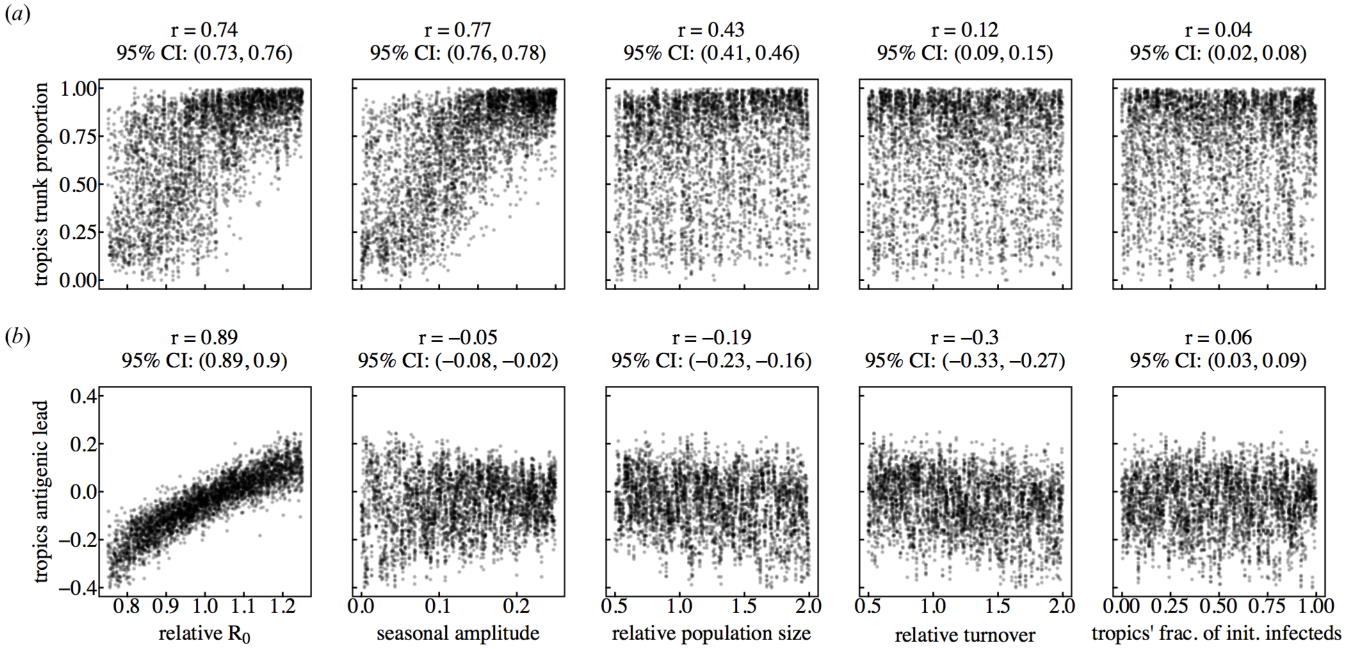

Figure S2: Multivariate sensitivity analysis showing effects of individual parameters on the (*a*) antigenic lead and (*b*) the fraction of the phylogenetic trunk in the tropics. Horizontal axes are projections of a Latin hypercube with dimensions corresponding to the five parameters indicated. Each point shows the mean value over a single simulation. The dashed lines represent the null hypotheses where (*a*) the trunk is distributed equally among the three regions or (*b*) tropical strains are neither antigenically ahead or behind. Pearson's correlation coefficients and associated 95% confidence intervals are indicated.

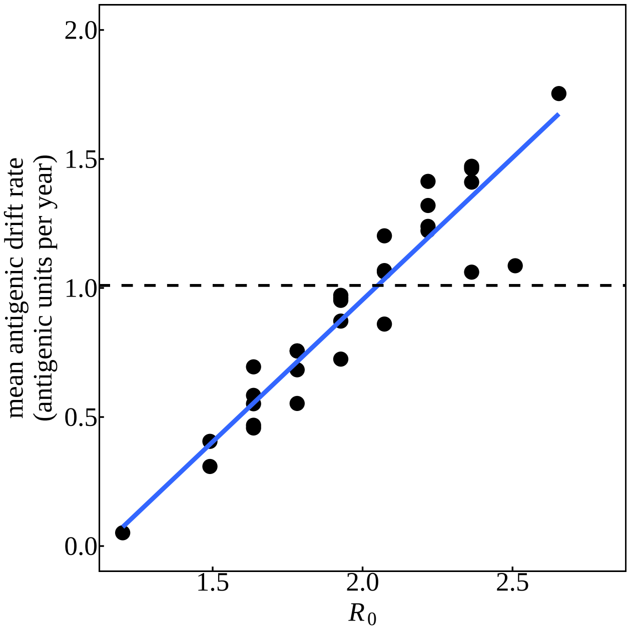

Figure S3: Effect of R_0 on antigenic drift in a single deme. Each point shows the mean antigenic drift rate from a single simulation. The blue line represents linear least squares regression, and the dashed line indicates the empirical estimate of the rate of antigenic drift for H3N2 [10]. Pearson's $r = 0.94$, $p < 0.001$; 95% CI: (0.88, 0.97).

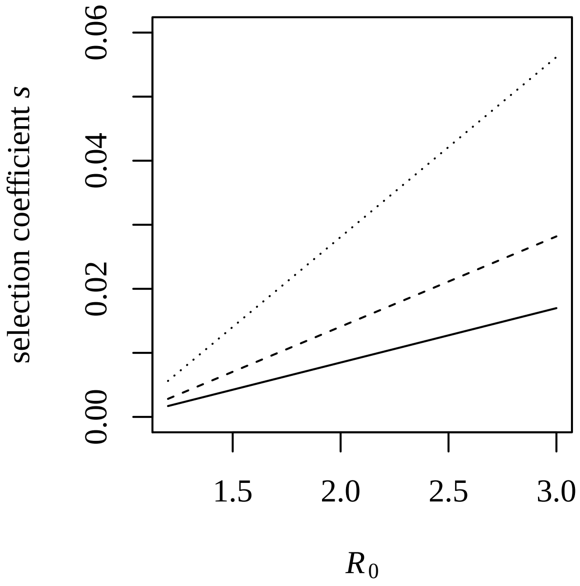

Figure S4: Relationship between R_0 and the selection coefficient for an invading strain with the resident at endemic equilibrium. The relationship for three different antigenic distances d between the invading strain and the resident strain is shown: 0.6 (solid line), 1.0 (dashed line), or 2.0 (dotted line) antigenic units.

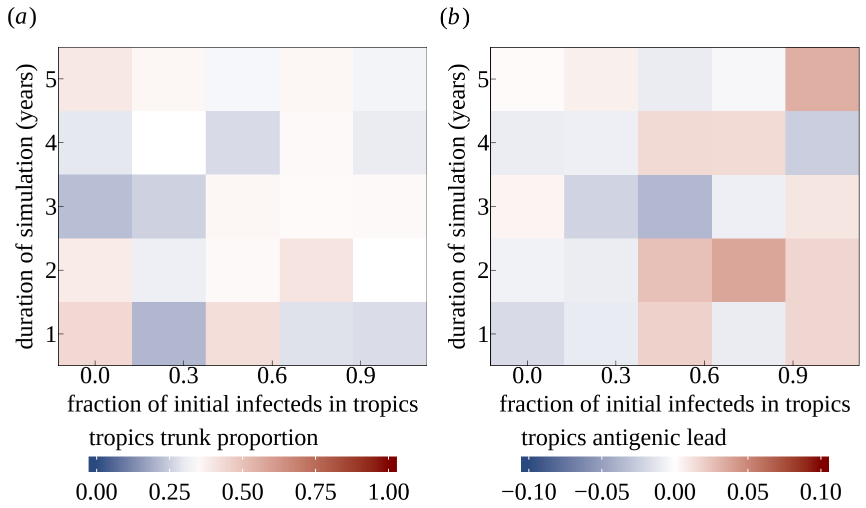

Figure S5: Effect of initial conditions on the antigenic lead and the fraction of the trunk in the tropics early in the simulation. Each square represents the average value for n=5 to 11 replicate simulations.

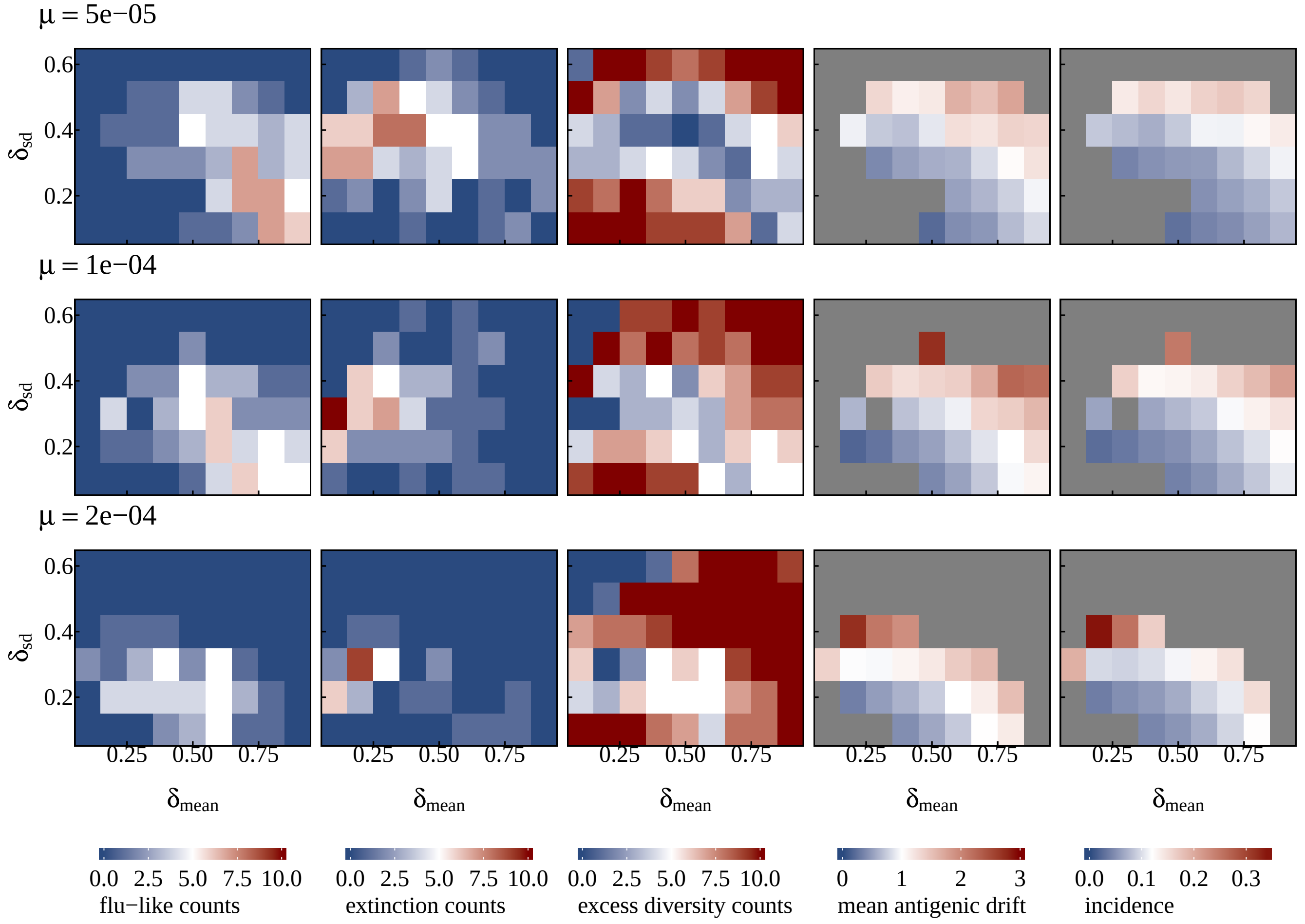

Figure S6: Sensitivity of influenza-like behaviour to changes in the mutational parameters, the mutation rate μ, mean mutation size δ_{mean}, and standard deviation of the mutation size δ_{sd}. Within each plot, each square represents ten replicate simulations. Each row of plots shows results from simulations using different mutation rates μ. The number of simulations where the virus went extinct is shown in the second column of plots, and the number of simulations where the viral population exceeded a TMRCA of 10 years is shown in the third column of plots. The remaining simulations are considered influenza-like and are shown in the first column of plots. The reported mean antigenic drift rates and prevalences are averaged over the influenza-like replicates. The color scales for mean antigenic drift and incidence are centered (white) at the observed values for H3N2 (table 1).

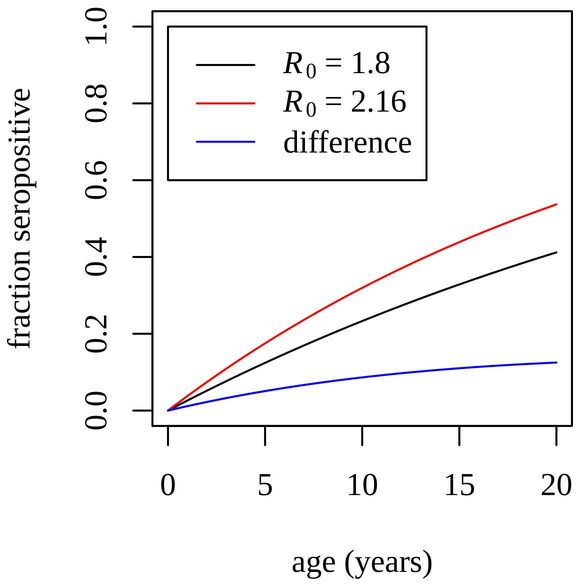

Figure S7: Theoretical increase in the fraction of seropositive individuals with age with $R_0 = 1.8$ and a 20% higher $R_0 = 2.16$.

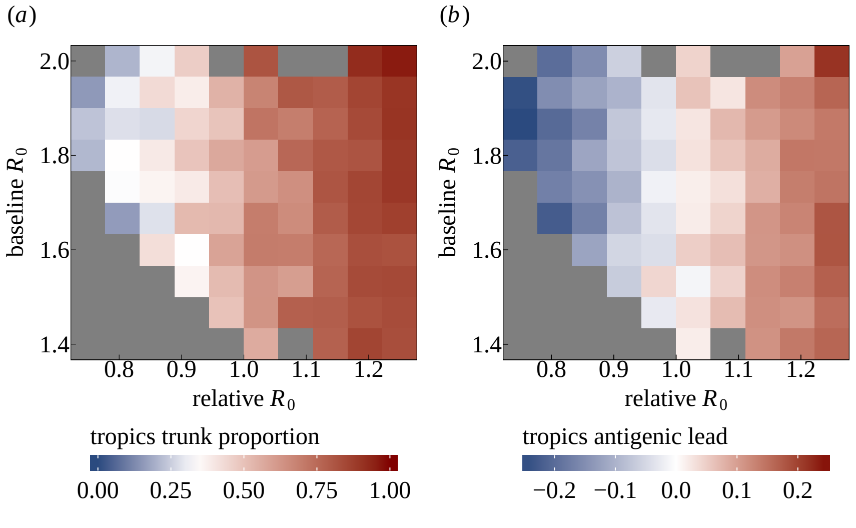

Figure S8: Lowering baseline R_0 decreases the effect of relative R_0 on the fraction of the trunk and antigenic lead in the tropics. (*a*) Effects of baseline and relative R_0 on the fraction of the trunk in the tropics. Blue indicates that the phylogenetic trunk is located in the tropics less than 1/3 of the time, and red indicates that the trunk is the tropics more than 1/3 of the time. (*b*) Effects of baseline R_0 and relative R_0 on antigenic lead in the tropics. Blue indicates that tropical strains are on average ahead antigenically relative to other strains, and red indicates that tropical strains are behind antigenically. Each square represents an average from 1 to 14 replicate simulations. Grey squares indicate parameter combinations where all of twenty attempted simulations either went extinct or exceeded the TMRCA threshold of 10 years.

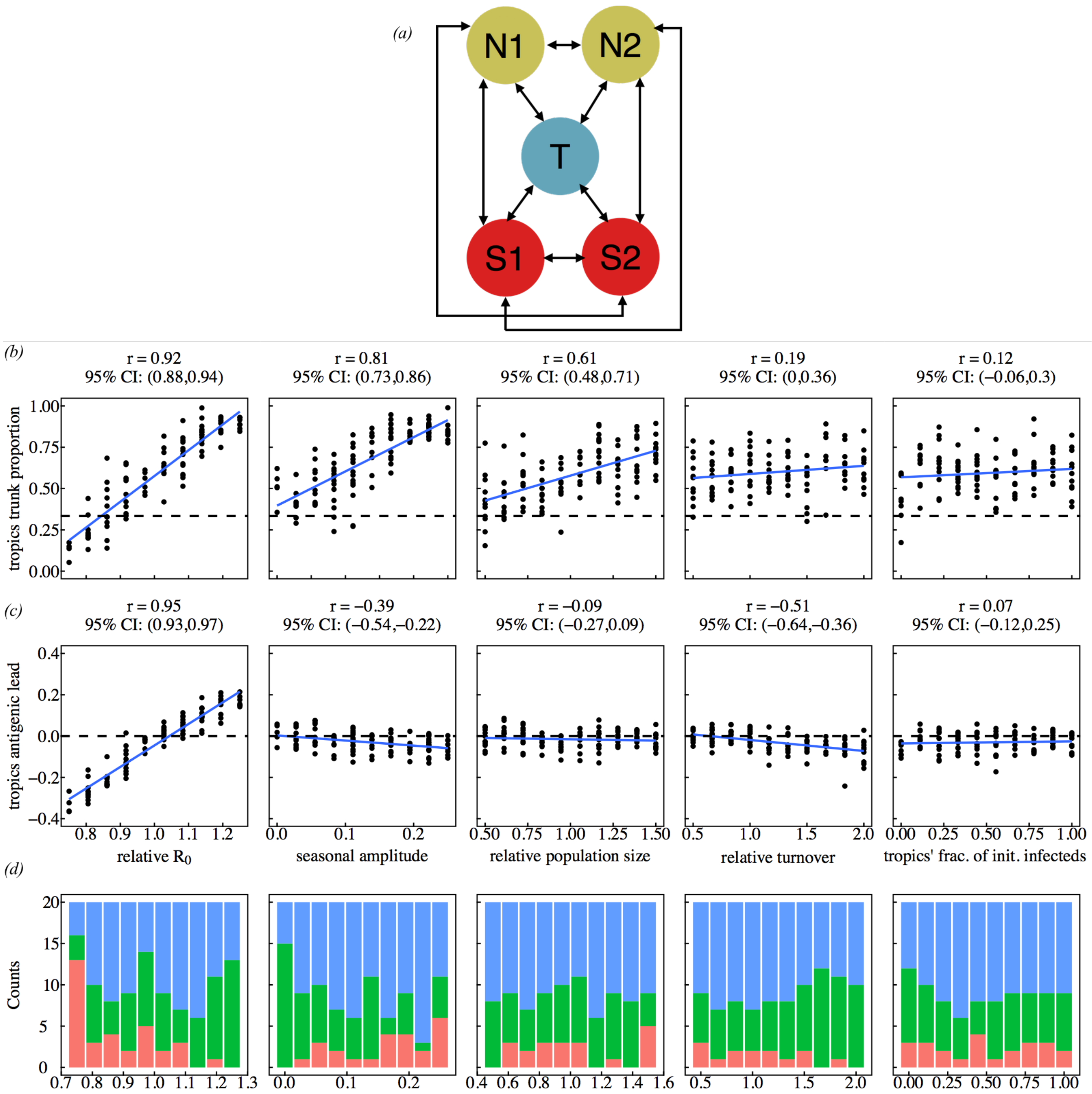

Figure S9: Univariate sensitivity analysis using a fully connected 5-deme model (a) showing the effects of individual parameters on (b) the antigenic lead and (c) the fraction of the phylogenetic trunk in the tropics. By default, the tropics have a population size that is twice as large as any single temperate deme. In each column of plots, only the parameter indicated on the x-axis is varying; all others are held constant at the default value. Each point represents the mean value over a single simulation. Blue lines indicate linear least squares regression. The dashed lines represent the null hypotheses where (b) the trunk is distributed proportionally to the default population size among the regions or (c) tropical strains are neither antigenically ahead or behind. (d) Number of simulations that went extinct (red), exceeded the TMRCA limit (green), or were suitable for analysis (blue).

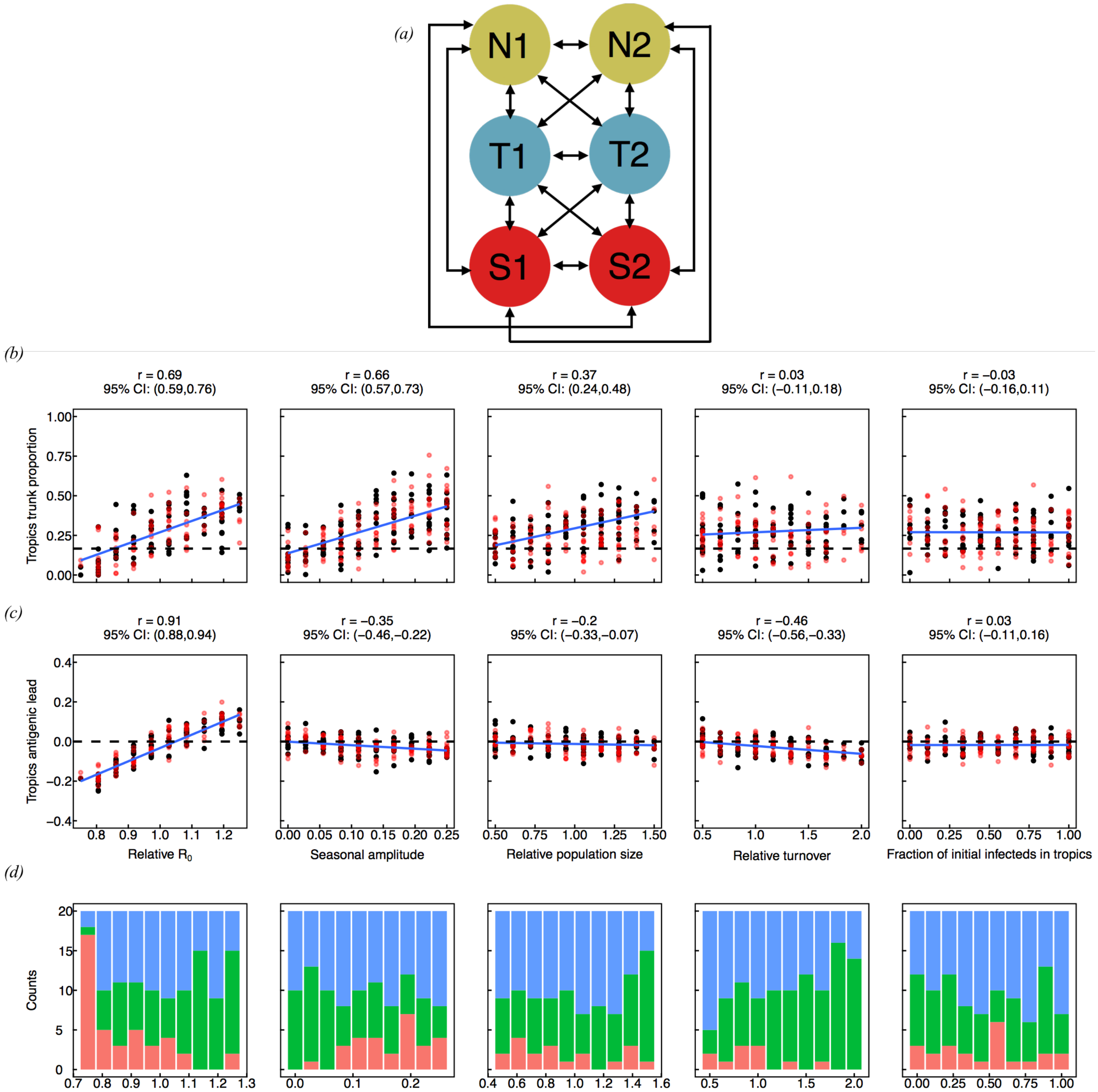

Figure S10: Univariate sensitivity analysis using a fully connected 6-deme model (*a*) showing the effects of individual parameters on (*b*) the antigenic lead and (*c*) the fraction of the phylogenetic trunk in each of the two tropical demes. By default, all demes have the same population size. In each column of plots, only the parameter indicated on the x-axis is varying; all others are held constant at the default value. Each point represents the mean value over a single simulation. Black points show results from one tropical deme and red points from the other. Blue lines indicate linear least squares regression to the combined data from both tropical demes. The dashed lines represent the null hypotheses where (*b*) the trunk is distributed proportionally to the default population size among the regions or (*c*) tropical strains are neither antigenically ahead or behind. (*d*) Number of simulations that went extinct (red), exceeded the TMRCA limit (green), or were suitable for analysis (blue).
